# Successful Smoking Cessation among Women Smokers Based on Utilizing National Smoking Cessation Service Type in Korea

**DOI:** 10.3390/ijerph18126578

**Published:** 2021-06-18

**Authors:** Dahyeon Lee, Kang-Sook Lee, Ahnna Lee, Hyeju Ahn, Hyun-Kyung Lee, Hyekyeong Kim, Jakyoung Lee, Hong-Gwan Seo

**Affiliations:** 1 Department of Health Promotion, Graduate School of Public Health, The Catholic University of Korea, Seoul 06591, Korea; dada2020@catholic.ac.kr; 2 Department of Preventive Medicine, College of Medicine, The Catholic University of Korea, Seoul 06591, Korea; abcde@catholic.ac.kr; 3 Department of Public Health, Graduate School, The Catholic University of Korea, Seoul 06591, Korea; 4 Korean Association on Smoking or Health, Seoul 07238, Korea; heyjude@kash.or.kr (H.A.); lovhyun@kash.or.kr (H.-K.L.); hkkim@ewha.ac.kr (H.K.); jakyl00722@gmail.com (J.L.); 5 Department of Health Convergence, Ewha Womans University, Seoul 03760, Korea; 6 Graduate School of Public Health, Yonsei University, Seoul 03722, Korea; 7 National Cancer Center, Goyang-si 10408, Korea

**Keywords:** smoking rate, women smokers, smoking cessation services, emotional labor workers

## Abstract

Background: This study aimed to evaluate the successful smoking cessation across different national smoking cessation services. Methods: This study included data that had been previously entered into the integrated information system for smoking cessation services and comprised 144,688 participants after excluding missing data. These clinics provide face-to-face counseling, phone calls, text messages, and e-mail services for six months and nine sessions. Results: The women-only program had the lowest success rate (11.3%). Compared with the women-only program, the six-month success rate of smoking cessation clinic at public health centers (OR = 3.72, CI = [3.52, 3.92]), visiting-type smoking cessation clinics (OR = 2.97, CI = [2.79, 3.16]), the residential 4 -night 5-day program (OR = 7.79, CI = [6.49, 9.35]), and a program for inpatients (OR = 2.36, CI = [1.89, 2.94]) showed a significant increase. Conclusions: Emotional labor workers who participated in the women-only program had low smoking cessation success rates, while those who participated in the residential 4-night 5-day program had high success rates.

## 1. Introduction

South Korea has begun to provide free smoking cessation counselling and treatment services at public health centers across the country. The government raised cigarette prices from 2000 to 2500 won (from $1.8 to $2.3) and has operated smoking cessation service centers in 254 public health centers since 2005. Local resources, including companies and schools, endorse diverse smoking cessation projects to reduce smoking rates and to improve the residents’ health. Smoking cessation projects implemented by the public health center include smoking cessation clinics, moving smoking cessation service, and campaigns.

After the cigarette price was hiked from 2500 to 4500 won (from $2.3 to $4.1) in 2015, the government initiated several visiting smoking cessation services, a residential 4-night 5-day program in 17 smoking cessation support centers, and hospital-based smoking cessation treatments. The visiting smoking cessation service provides customized services for those who are facing a smoking-related blind spot. The term “smoking blind spot” means a class belonging to a group that does not voluntarily come to a smoking cessation clinic and lacks access to smoking cessation clinics. These individuals include out-of-school youths, college students, women, and disabled persons, who often experience difficulties in visiting public health centers [1].

Consequently, the male smoking cessation rate dropped from 51.7% to 39.4% between 2005 and 2015. The National Health and Nutrition Examination Survey, which was conducted by the Korea Center for Disease Control and Prevention, shows that the female smoking rate increased from 5.5% to 7.5% between 2015 and 2018. This trend has been especially noticeable among young people. Among women in their 20s, the smoking rate is from 6.9% to 10.9%, and from 6.7% to 8.3% among women in their 30s. The average smoking initiation age decreased from 29.4 to 23.8 years between 1998 and 2018 [2]. This trend is also seen in the United States [3].

Several factors can affect women’s smoking [4]. In Korea, it is a social atmosphere in which women should not be exposed to smoking. In Korea, there is an atmosphere in which smoking women are branded as immoral. Therefore, most smoking women hide the fact that they smoke. The smoking reporting rate may be low among users living in local communities with high sociocultural pro-smoking pressure considering this social atmosphere, thus South Korean women’s actual smoking rate is likely to be much higher than the reporting rate [5,6]. In fact, the female smoking rate drawn from the cotinine value, which is a nicotine metabolite, was reported to be 2.36 times higher than the self-reported measurement-based smoking rate [7].

Women smokers often find it difficult to quit smoking because of such social effects, and they often have no close supporters to aid them in their smoking cessation efforts. Even if women smokers experience withdrawal symptoms, they may find it difficult to request help from others [8,9]. These social effects can influence women smokers to become passive toward direct smoking cessation counseling [10].

The Korean government is currently providing gender-neutral national smoking cessation services, but it is also separately implementing a women-only program. In particular, the smoking rate tends to be higher among emotional labor workers, such as telemarketers and salesclerks, who are more prone to illness. Thus, the government is providing them with smoking cessation services [11]. Emotional labor is paid labor, mainly to express or suppress emotions, and to manage and control emotions for one’s duties. Emotional workers are mostly women in these professions [12,13].

While their overall smoking cessation attempt rate is higher than that of men, women form a comparatively low percentage (10.2%) of actual users of smoking cessation clinics in public health centers [14]. The characteristics and smoking cessation success rates of women users of smoking cessation services must be discussed. Even when these women quit smoking, their smoking cessation maintenance rate tends to be low in the long run [15].

Numerous studies have examined the development of smoking cessation program and verified their effectiveness, but no research has examined women smokers’ characteristics and smoking cessation success rates based on the type of smoking cessation service they use. This study aimed to evaluate the successful smoking cessation across different national smoking cessation services.

## 2. Materials and Methods

### 2.1. Participants

This study included data that had been previously entered into the integrated information system for smoking cessation services (nosmk.khealth.or.kr) and are based on the counseling records of 172,559 female users of the national smoking cessation service between 1 January 2017 and 31 December 2019. We used 144,688 participants after excluding missing data from 12,300 people from smoking cessation clinic in public health centers (11.51%), 8543 from moving smoking cessation clinic (27.73%), 5268 from campaign-type smoking cessation clinic (46.47%), 119 from residential 4-night 5-day program (18.98%), 103 from program for inpatient (18.39%), 422 from program for out-of-school youths (9.19%), 858 from women-only program (5.81%), 189 from program for college students (7.42%), and 69 from program for disabled persons (14.87%). This study was approved by the Institutional Review Board of the Catholic University of Korea (MC20ZASI0158). This study used data from smoking cessation clinics in 254 public health centers and 17 regional smoking cessation support centers. They are open to any smoker in the local community, regardless of gender.

### 2.2. The Process of Various Programs

These clinics provide services such as smoking cessation counseling or cotinine measurement, smoking cessation supplements (behavior-enhancing items; tooth-brushing set, mouthwash, mint candies, and grip strength systems), behavioral therapy, and smoking cessation treatments (nicotine replace therapy; nicotine patches, chews, prescription medication; Bupropion, Vareniclien). When a potential patient registers at a smoking cessation clinic, they can receive counseling only after they have provided the necessary consent for their personal information to be processed and have agreed to the provision of their personal information to third parties under the Privacy Act. These clinics provide face-to-face counseling, phone calls, text messages, and e-mail services for up to six months for nine sessions from the start of the smoking cessation program. Public health centers can also be visiting-type clinics for employees of large companies and campaign-type clinics, which are held outside and promote smoking cessation clinics.

The government regional smoking cessation support center aims to increase smoking cessation attempts and practice rates and to ease smoking rates among vulnerable populations by providing visiting smoking cessation services to out-of-school youths, women, college students, disabled persons, and small business workers. Moving smoking cessation service is provided by visiting the relevant place in connection with the business or school. Visiting smoking cessation services promote and provide smoking cessation services by visiting blind spot to those who lack access to smoking cessation services.

The women-only program is intended mainly to serve those with severe stress from emotional labor and in sales. Moreover, regional smoking cessation support centers systematically and professionally implement the residential 4-night 5-day program with medication for heavy smokers, repeated relapsers, and acute and chronic disease patients, who require urgent smoking cessation aid. This can help increase smoking cessation attempts and practice rates and reduced smoking rates. It also provides smoking cessation services to inpatients at hospitals.

### 2.3. Measurement

#### 2.3.1. General Characteristics

The general characteristics of the selected participants were age, occupation, exercise, and alcohol use. The age assessment was categorized based on generations and divided based on every 10 years. Regarding the occupational categories, managers, professionals, and office workers were classified as “office workers”; sales and service workers as “service workers”; workers in agriculture and fisheries, equipment and machinery workers, technicians, and simple labor workers as “functional workers”; service person, college students, and workers having other occupations as “others”; and homemakers and unemployed workers as “unemployed”. The questionnaire regarding alcohol determined whether the participant had been drinking for the past year using yes–no questions. The questionnaire regarding exercise status asked users whether they had exercised over the past year using yes–no questions.

#### 2.3.2. Smoking-Related Characteristics

The smoking-related characteristics included CO expiration (ppm), average daily amount of smoking, smoking initiation age, total smoking period, and smoking cessation attempt period. The average daily amount of smoking was measured based on the number of cigarettes, the total smoking period using years, and the smoking cessation attempt period using days.

#### 2.3.3. Smoking Cessation Success Rate

The smoking cessation service conducted a smoking cessation success assessment for those who started cessation attempts after four different durations: four weeks, six weeks, twelve weeks, and six months. The following criteria indicated successful smoking cessation: self-report, CO level measurements, and cotinine level measurements. Any participant who failed to maintain smoking cessation midway through the six months, expressed an intention to suspend or refuse further participation, or lost contact with the program was classified as a smoking cessation failure.

### 2.4. Statistical Analysis

We used the SPSS 27.0 statistics program for the analysis. The general characteristics of individuals who used each smoking cessation service and the success rates for each period—four weeks, six weeks, twelve weeks, and six months—were assessed using a chi-square test. Analysis of variance (ANOVA) and Scheffe analyses were conducted to identify smoking-related characteristics. Regarding the period-based success rate analysis for each smoking cessation service, a logistic regression was conducted by adjusting the age, exercise status, and alcohol use of each participant. The women-only program was used as the reference group.

## 3. Results

### 3.1. Participant Characteristics

Of all the selected assessed age groups, those in their 20s formed the largest group of smokers (19.51%). Regarding occupation, “others” formed the largest category (33.88%), followed by “unemployed” (19.29%) and “service workers” (17.64%).

Participants in the program for college students (65.46%) formed the highest percentage among those who answered “yes” to the question regarding whether they had drunk alcohol in the past year, followed by participants in the out-of-school youth program (57.15%). However, 5694 people in the campaign-type smoking cessation clinics (98.29%) formed the highest proportion of those who said that they had not drunk alcohol in the past year.

Participants in the residential 4-night 5-day program formed the highest proportion of those who answered “yes” to the question regarding whether they had exercised in the past year (14.34%), while participants in the campaign-type smoking cessation clinics formed the lowest percentage (0.49%) (Table 1).

### 3.2. Smoking-Related Characteristics

The overall average of CO expiration was 8.60 ppm, with the average of the group with the lowest rate being 3.48 ppm in campaign-type smoking cessation clinics. The total average daily amount of smoking was 12.18 cigarettes. People in the residential 4-night 5-day program smoked the most at 18.25 cigarettes.

The total average smoking initiation age was 22.83 years. Excluding the program for out-of-school youths and for college students, the participants in the women-only program had the lowest average smoking initiation age at 20.52 years.

The total average smoking period was 18.20 years. The group in the residential 4-night 5-day program showed the longest smoking period: 28.54 years. The total average period of smoking cessation attempts was 29.60 days. In this regard, participants in the program for disabled persons had the longest period at 49.56 days (Table 2).

### 3.3. Success of Smoking Cessation Based on Duration

The group with the highest success rate was the residential 4-night 5-day program with durations starting from four weeks onward (90.7%). The women-only program had the highest failure rate and lowest success rate (11.3%) for a period of six months. The total success rate for four weeks was 90,765 (62.7%), but the total success rate for 6 months was 40,728 (28.1%). This figure dropped steeply from four weeks onward (Table 3). The success rate did not differ significantly between before and after excluding the missing data.

## 4. Odds Ratios for Success of Smoking Cessation According to Each Smoking Cessation Service Compared with the Women-Only Program

Compared with the women-only program, participants in the smoking cessation clinics at public health centers (OR = 4.06, CI = [3.91, 4.22]), visiting-type smoking cessation clinics (OR = 3.82, CI = [3.65, 4.00]), residential 4-night 5-day program (OR = 16.02, CI = [11.84. 21.68]), program for inpatients (OR = 2.55, CI = 2.11–3.09), and program for disabled persons (OR = 1.35, CI = 1.10–1.65) showed significant results at the four-week success rate. The success rates for the six-week and twelve-week durations also showed similar patterns. Regarding the six-month success rate, smoking cessation clinics at public health centers (OR = 3.72, CI = [3.52, 3.92]), visiting-type smoking cessation clinics (OR = 2.97, CI = [2.79, 3.16]), residential 4-night 5-day program (OR = 7.79, CI = [6.49, 9.35]), and program for inpatients (OR = 2.36, CI = [1.89, 2.94]) showed significant results (Table 4).

## 5. Discussion

This study showed that most women smokers using the national smoking cessation service were in their 20s. This finding agrees with national statistics, which have shown that the smoking initiation age for women is dropping. Women in their 20s are young, so smoking during this period may not only affect their current health, but may affect future health considerations such as childbirth and parenting [16].

Among the occupational categories, “others” was the highest, and mainly service workers and unemployed including homemakers. Service workers tend to smoke frequently because they are mostly engaged in emotional labor. In particular, female sales workers are often exposed to numerous job stressors, including emotional stress, workplace harassment, and job instability [13]. Call center workers also tend to suffer from high stress levels [17]. Environments that could endanger workers’ mental health tend to make smoking cessation more difficult. Another study involving call center workers found that non-smokers tend to engage in other activities to improve their health, but that people who have high stress levels tend to smoke to ease their work-related emotional burdens. In this study, the high smoking rates among female call center staff could be attributed to the job culture in call centers [18].

Smoking cessation services are provided as a women-only program, but the cessation success rates for female emotional labor workers are lower than that of all other women workers. Implementing and improving smoking cessation services is important for emotional labor groups. Most emotional workers work as non-regular workers and dispatched workers, so they have short breaks at work and a lack of rest areas, so they cannot be used freely. As such, emotional workers are socially and economically vulnerable. A study showed that smoking rates vary based on industry and occupation and the causes of each smoking behavior may vary, so diverse smoking cessation programs must be developed to reduce overall smoking rates [19].

The proportion of participants that answered yes if they had drunk in the past year was higher in the college student program and the out-of-school youths program than in other programs [20]. As successful smoking cessation may lead to rising drinking rates, implementing drinking cessation education alongside the smoking cessation program may be necessary [21].

Groups with a high percentage of people who have not consumed any alcohol over the past year can use visiting-type program and campaign-type program, as these do not have a high success rate for smoking cessation. A previous study has shown that non-drinkers are also unlikely to achieve early smoking cessation [21].

The question regarding participants’ exercise status over the past year showed that participants in the residential 4-night 5-day program had the highest percentage of positive answers, indicating that they were highly interested in their own health and were ready to quit smoking.

Regarding the smoking initiation age, participants in the women-only program had the earliest initiation ages (20.52 years), with those in out-of-school youth program and college student program being the exception to this trend. Thus, smoking cessation education for women should start during their adolescent years. The social atmosphere surrounding teenagers and young people could affect their initiation and cessation behaviors with regard to smoking. For this age group, smoking cessation education may be more effective if it focuses on smoking in peer groups [22].

Despite having the highest CO expiration level (10.30 ppm), the highest average daily smoking (18.25 cigarettes), and the longest total smoking period (28.54 years), participants in the residential 4-night 5-day program had the highest success rate. This shows that, even when the smoking period is long and the average daily smoking amount is large, providing appropriate smoking cessation services with medication treatment for four days and five nights in the hospital can lead to high success rates.

The average smoking cessation attempt duration was 29.60 days, while that of participants in the women-only program was 36.18 days—higher than for all other participants—indicating that they are willing to attempt cessation.

An examination of the success rates for the period of four weeks to six months shows that participants in the residential 4-night 5-day program group had the highest success rates. Prior studies have also shown that smokers who participated in the residential smoking cessation program continue to have longer smoking cessation effects than smokers who have experienced other programs [23]. A previous study also demonstrated that a residential smoking cessation program is effective for heavy smokers who are highly dependent on nicotine [24]. This is because the residential smoking cessation program provides various combined interventions, including medication treatment, psychological counseling, exercise prescription, and nutrition counseling, to vulnerable smokers [25]. To successfully achieve smoking cessation and maintain it, participants must develop a strong motivation to work toward smoking cessation. The participants in the residential 4-night 5-day program have shown motivation and sufficient preparation to participate in a program where they would have to spend four nights and five days in a hospital. However, emotional labor workers, who tend to be the main users of the women-only program, do not have strong motivation regarding smoking cessation [26].

Because women engaged in emotional labor and female college students have high smoking rates, a smoking cessation camp for women, which could run from Friday to Sunday, should be developed urgently. Prior studies have also demonstrated that a three-day residence program is more cost-effective than a long-term residence program. Exclusive smoking cessation camps should be conducted for women [27]. This will help mitigate any barriers they may face in terms of entering treatment. For example, there are temptations from co-workers, social atmosphere in which they should not be caught smoking, and work stress. Therefore, users’ preferences can be better met when providing women-only services [28].

In the inpatient program, which is also a residential treatment program, when a patient wants to use the smoking cessation service and expresses one’s intention to the bedside doctor, they are allowed to ask for cooperation from the doctors at the smoking cessation center. Medical staff from the smoking cessation center will then visit the patient to provide smoking cessation counseling and prescribed medication for six months, and counselors will conduct counseling for the next six months. A study recommends further expanding this medical approach to encourage patients who visit the emergency room to ask questions about the smoking cessation service [29]. Low-income individuals may have fewer opportunities to receive help from experts, so informing all patients in each emergency room about the smoking cessation services could help extend the opportunity to quit smoking to smokers from all social backgrounds.

Smoking cessation clinics at public health centers also tend to have higher success rates compared with the women-only program. People tend to deliberately and directly visit such clinics, while the women-only program is often conducted by visiting potential smokers’ workspaces, which are likely to have high-stress working conditions for female workers who participate involuntarily. The users of the women-only program may be less prepared to quit smoking [30].

Out-of-school youths form another vulnerable smoker population, as they tend to face a mixture of problems, including drinking and academic performance pressure, as well as smoking. It may be difficult for this population to think of smoking as an immediate concern, and they may show little or no interest in smoking cessation. A previous study suggests that behavior reinforcement through group counseling is appropriate for adolescents because their smoking or non-smoking behaviors are often influenced by their peers [31]. The out-of-school youth program is currently testing customized counseling. Follow-up research is necessary to determine whether group counseling could fit the reality of South Korea. This study had some limitations. The data were sourced from self-reported answers for the surveys, the nature of the survey implied the possibility of understatement, and it was difficult to know the characteristics of each smoker. Furthermore, this study’s findings had limited generalizability because it was conducted among national smoking cessation service users in Korea. In addition, medication usage was not considered in this study. The other limitation was that the number of variables was limited because this study used secondary data, and the association of variables (socio-economy characteristics with education attainment). The long-term follow-up study should be conducted in women who used various national smoking cessation services.

## 6. Conclusions

The government of Korea operates a women’s smoking cessation service targeting emotional labor workers.

However, the decrease in women smoking rates is small. No previous research has examined women smokers’ characteristics and smoking cessation success rates based on the type of smoking cessation service they use.

The findings of this study have identified the characteristics of women who use smoking cessation services and specifically analyzed the success rate for each service. A very intensive residential program for women must be developed in the future. Emotional labor workers who participated in the women-only program had low smoking cessation success rates, while those who participated in the residential 4-night 5-day program had high success rates. Emotional laborers who are trying to quit smoking should be assisted by their employers by reducing and resolving workplace stress, improving working conditions, and providing company support when required. Exclusive residential programs should be implemented for women.

## Figures and Tables

**Table 1:** General characteristics of study subjects according to various smoking cessation programs.

	Public Health Center			Residential Program		Visiting Smoking Cessation Service					
Variable	Smoking Cessation Clinic *n* = 94,562 (%)	Moving Smoking Cessation Service *n* = 22,270 (%)	Campaign *n* = 6068 (%)	4-Night 5-Day Program *n* = 508 (%)	Inpatient Program *n* = 457 (%)	Out of School Youth Program *n* = 4168 (%)	Women-Only Program *n* = 13,903 (%)	College Students Program *n* = 2357 (%)	Disabled Program *n* = 395 (%)	Total *n* = 144,688 (%)	*p*-Value
Age											
10–19	7238 (7.7)	5376 (24.1)	781 (12.9)	0 (0.0)	17 (3.7)	4034 (96.8)	1218 (8.8)	1012 (42.9)	11 (2.8)	19,687 (13.6)	0.001
20–29	15,353 (16.2)	4563 (20.5)	956 (15.8)	5 (1.0)	51 (11.2)	134 (3.2)	5800 (41.7)	1331 (56.5)	30 (7.6)	28,223 (19.5)	
30–39	18,200 (19.3)	2404 (10)	538 (8.9)	76 (15.0)	65 (14.2)	0 (0.0)	3136 (22.6)	12 (0.5)	67 (17.0)	24,498 (16.9)	
40–49	19,756 (20.9)	2826 (12.7)	872 (14.4)	119 (23.4)	110 (24.1)	0 (0.0)	2188 (15.7)	2 (0.1)	95 (24.1)	25,968 (18.1)	
50–59	20,182 (21.3)	3274 (14.7)	1150 (19.0)	178 (35.0)	113 (24.7)	0 (0.0)	1098 (7.9)	0 (0.0)	95 (24.1)	26,090 (18.0)	
60–	13,833 (14.7)	3827 (12.2)	1771 (29.2)	130 (25.6)	101 (22.1)	0 (0.0)	463 (3.3)	0 (0.0)	97 (24.5)	20,222 (14.0)	
Job											
Office	7461 (7.9)	1706 (7.7)	135 (2.2)	50 (9.8)	22 (4.8)	0 (0.0)	934 (6.7)	1 (0.0)	9 (2.3)	10,318 (7.1)	0.001
Service	16,907 (17.9)	2142 (9.6)	447 (7.4)	54 (10.6)	75 (16.4)	0 (0.0)	5876 (42.3)	3 (0.1)	13 (3.3)	25,517 (17.6)	
Skilled	3884 (4.1)	716 (3.2)	62 (1.0)	14 (2.8)	11 (2.4)	0 (0.0)	873 (6.3)	0 (0.0)	10 (2.5)	5570 (4.0)	
Etc.	30,482 (32.2)	8557 (38.4)	3516 (57.9)	153 (30.1)	83 (18.2)	0 (0.0)	3928 (28.3)	2248 (95.4)	49 (12.4)	49,016 (33.9)	
No job	23,439 (24.8)	2508 (11.3)	446 (7.4)	179 (35.2)	179 (39.2)	0 (0.0)	943 (6.8)	0 (0.0)	213 (53.9)	27,907 (19.3)	
Youth	5145 (5.4)	2729 (12.3)	261 (4.3)	0 (0.0)	0 (0.0)	4168 (100)	1 (0.0)	1 (0.0)	0 (0.0)	12,305 (8.5)	
No Response	7244 (7.7)	3912 (17.6)	1201 (19.8)	58 (11.4)	87 (19.0)	0 (0.0)	1348 (9.7)	104 (4.4)	101 (25.6)	14,055 (9.7)	
Drink											
Yes	38,336 (40.5)	3874 (17.4)	104 (1.7)	223 (43.9)	171 (37.4)	2382 (57.2)	6029 (43.4)	1543 (65.5)	103 (26.1)	52,765 (36.5)	0.001
No	56,226 (59.5)	18,396 (82.6)	5964 (98.3)	285 (56.1)	286 (62.6)	1786 (42.9)	7874 (56.6)	814 (34.5)	292 (73.9)	91,923 (63.5)	
Exercise											
Yes	21,768 (23.0)	1842 (8.3)	30 (0.49)	210 (41.3)	78 (17.1)	865 (20.8)	2238 (16.1)	545 (23.1)	147 (37.3)	27,723 (19.2)	0.001
No	72,794 (77.0)	20,428 (91.7)	6038 (99.51)	298 (58.7)	379 (82.9)	3303 (79.3)	11,665 (83.9)	1812 (76.9)	248 (62.8)	116,965 (80.8)	

**Table 2:** Smoking-related characteristics of study subjects.

	Public Health Center			Residential Program		Visiting Smoking Cessation Service					
Variable	Smoking Cessation Clinic (*n* = 94,562)	Moving Smoking Cessation Service (*n* = 22,270)	Campaign (*n* = 6068)	4-Night 5-Day Program (*n* = 508)	Inpatient Program (*n* = 457)	Out of School Youth Program (*n* = 4168)	Women-Only Program (*n* = 13,903)	College Students Program (*n* = 2357)	Disabled Program (*n* = 395)	Total (*n* = 144,688)	*p*-Value
	Mean ± SD	Mean ± SD	Mean ± SD	Mean ± SD	Mean ± SD	Mean ± SD	Mean ± SD	Mean ± SD	Mean ± SD	Mean ± SD	
Cigarettes per day	13.8 (8.2) ^e^	7.9 (6.9) ^b^	5.7 (5.2) ^a^	18.3 (7.9) ^g^	15.5 (8.5) ^f^	13.8 (9.4) ^e^	10.3 (6.4) ^c^	9.6 (5.9) ^c^	12.1 (9.8) ^d^	12.2 (8.2)	0.001
Age of first smoking	23.8 (8.4) ^e^	21.9 (7.5) ^d^	24.6 (8.1) ^e^	24.7 (7.8) ^e^	25.1 (8.7) ^f^	14.4 (1.6) ^a^	20.5 (6.2) ^c^	17.2 (2.4) ^b^	24.9 (8.9) ^ef^	22.8 (8.1)	0.001
Smoking period	19.7 (11.5) ^d^	17.3 (14.8) ^c^	22.2 (15.8) ^e^	28.5 (8.4) ^g^	23.4 (11.9) ^ef^	3.4 (1.8) ^a^	13.5 (9.6) ^b^	4.5 (2.7) ^a^	24.7 (12.0) ^f^	18.2 (12.5)	0.001
Quit smoking attempt period	34.3 (147.4) ^c^	11.8 (74.3) ^ab^	1.1 (17.1) ^a^	24.4 (109.1) ^b^	33.5 (158.2) ^bc^	31.4 (87.6) ^b^	36.2 (148.9) ^c^	38.6 (106.2) ^cd^	49.6 (214.7) ^d^	29.6 (134.0)	0.001
CO level (ppm)	9.8 (8.8) ^ef^	5.2 (6.3) ^b^	3.5 (4.6) ^a^	10.3 (8.0) ^f^	6.3 (7.8) ^bc^	6.0 (6.7) ^b^	9.1 (7.8) ^e^	7.6 (6.8) ^cd^	8.8 (8.4) ^de^	8.6 (8.4)	0.001
4 weeks success	9.3 (8.6)	4.9 (6.1)	3.9 (5.2)	10.1 (7.8)	6.1 (7.7)	4 (5.9)	8.1 (7.6)	6.5 (6.8)	6.90 (8.6)		
failure	11.0 (9.2)	5.8 (6.6)	3.4 (4.6)	12.7 (9.3)	6.7 (7.9)	6.9 (6.8)	9.6 (7.8)	8.1 (6.7)	10.3 (7.9)		
6 weeks success	9.2 (8.5)	4.8 (6.0)	3.8 (4.6)	9.9 (7.8)	6.1 (7.8)	3.8 (5.7)	7.8 (7.6)	6.1 (6.6)	6.0 (7.8)		
failure	10.9 (9.1)	5.9 (6.6)	3.5 (4.6)	12.6 (8.6)	6.5 (7.8)	6.76 (6.8)	9.6 (7.8)	8.2 (6.8)	10.1 (8.4)		
12 weeks success	8.8 (8.4)	4.5 (5.9)	4.1 (5.2)	9.7 (7.9)	5.8 (8.0)	3.20 (5.4)	7.2 (7.5)	5.6 (6.6)	5.0 (7.7)		
failure	10.8 (9.1)	5.8 (6.6)	3.5 (4.6)	11.9 (8.1)	6.6 (7.7)	6.56 (6.7)	9.5 (7.8)	8.1 (6.8)	9.8 (8.3)		
6 months success	8.2 (8.1)	4.2 (5.5)	4.2 (5.3)	9.4 (7.9)	4.9 (7.0)	2.70 (4.9)	5.5 (6.3)	2.7 (4.9)	3.5 (6.4)		
failure	10.6 (9.0)	5.6 (6.5)	3.5 (4.6)	11.3 (7.9)	6.7 (8.0)	6.31 (6.7)	9.5 (7.8)	6.3 (6.7)	9.7 (8.4)		

*n* ^a–f^: same character was not significant difference by analysis of variance (ANOVA) Scheffe’s multiple comparison.

**Table 3:** Success rate according to various smoking cessation programs.

	Public Health Center			Residential Program		Visiting Smoking Cessation Service					
Variable	Smoking Cessation Clinic *n* = 94,562 (%)	Moving Smoking Cessation Service *n* = 22,270 (%)	Campaign *n* = 6068 (%)	4-Night 5-Day Program *n* = 508 (%)	Inpatient Program *n* = 457 (%)	Out of School Youth Program *n* = 4168 (%)	Women-Only Program *n* = 13,903 (%)	College Students Program *n* = 2357 (%)	Disabled Program *n* = 395 (%)	Total (*n* = 144,688)	*p*-Value
4 weeks											
Success	66,641 (70.5)	15,491 (69.6)	537 (8.8)	461 (90.7)	276 (60.4)	1318 (31.6)	5008 (36)	854 (36.2)	179 (45.3)	90,765 (62.7)	0.001
Failure	27,921 (29.5)	6779 (30.4)	5531 (91.2)	47 (9.3)	181 (39.6)	2850 (68.4)	8895 (64)	1503 (63.8)	216 (54.7)	53,923 (37.3)	
6 weeks											
Success	60,263 (63.7)	13,977 (62.8)	450 (7.4)	427 (84.1)	240 (52.5)	1107 (26.6)	4071 (29.3)	738 (31.3)	130 (32.9)	81,403 (56.3)	0.001
Failure	34,299 (36.3)	8293 (37.2)	5618 (92.6)	81 (15.9)	217 (47.5)	3061 (73.4)	9832 (70.7)	1619 (68.7)	265 (67.1)	63,285 (43.7)	
12 weeks											
Success	47,181 (49.9)	10,442 (46.9)	312 (5.1)	361 (71.1)	170 (37.2)	735 (17.6)	2766 (19.9)	485 (20.6)	87 (22)	62,539 (43.2)	0.001
Failure	47,381 (50.1)	11,828 (53.1)	5756 (94.9)	147 (28.9)	287 (62.8)	3433 (82.4)	11,137 (80.1)	1872 (79.4)	308 (78)	82,149 (56.8)	
6 month											
Success	31,631 (33.5)	6313 (28.3)	106 (1.7)	269 (53)	113 (24.7)	400 (9.6)	1567 (11.3)	270 (11.5)	59 (14.9)	40,728 (28.1)	0.001
Failure	62,931 (66.5)	15,957 (71.7)	5962 (98.3)	239 (47)	344 (75.3)	3768 (90.4)	12,336 (88.7)	2087 (88.5)	336 (85.1)	103,960 (71.9)	

**Table 4:** Odds ratios for success of smoking cessation according to various smoking cessation program compared with the women-only program.

Type of National Smoking Cessation Service		4 Weeks	6 Weeks	12 Weeks	6 Month
		OR (95% CI)	OR (95% CI)	OR (95% CI)	OR (95% CI)
Public health center	Smoking cessation clinic	4.06 (3.91–4.22)	4.05 (3.89–4.21)	3.79 (3.63–3.96)	3.72 (3.52–3.92)
	Moving smoking cessation service	3.82 (3.65–4.00)	3.89 (3.71–4.08)	3.39 (3.22–3.56)	2.97 (2.79–3.16)
	Campaign	0.16 (0.14–0.18)	0.18 (0.16–0.20)	0.20 (0.17–0.22)	0.12 (0.10–0.15)
Residential program	4-night 5-day program	16.02 (11.84–21.68)	11.55 (9.08–14.69)	8.83 (7.26–10.76)	7.79 (6.49–9.35)
	Inpatient program	2.55 (2.11–3.09)	2.50 (2.07–3.02)	2.20 (1.81–2.67)	2.36 (1.89–2.94)
Visiting smoking cessation service	Out of school youth program	0.87 (0.81–0.94)	0.94 (0.87–1.02)	0.95 (0.87–1.04)	0.94 (0.84–1.06)
	College students program	1.07 (0.98–1.17)	1.18 (1.07–1.29)	1.14 (1.02–1.27)	1.13 (0.98–1.30)
	Disabled program	1.35 (1.10–1.65)	1.07 (0.87–1.33)	1.01 (0.79–1.29)	1.20 (0.91–1.60)
	Women-Only Program	1	1	1	1

Adjusted for age, drink, and exercise.

## Data Availability

The data presented in this study are available on request from the corresponding author. The data are not publicly available due to privacy.
